# Significance of DMBT1 in Papillary Thyroid Carcinoma Concurrent With Hashimoto’s Thyroiditis

**DOI:** 10.3389/fonc.2021.680873

**Published:** 2021-08-04

**Authors:** Xiao-xiong Gan, Ya-yi Li, Si-jin Li, Shi-sen Mo, Jian-hua Feng, Fei Shen, Wen-song Cai, Ye-qian Lai, Bo Xu

**Affiliations:** ^1^Department of Thyroid Surgery, Guangzhou First People’s Hospital, School of Medicine, South China University of Technology, Guangzhou, China; ^2^General Surgery, Guangzhou First People’s Hospital, School of Medicine, South China University of Technology, Guangzhou, China; ^3^Department of Thyroid Surgery, Meizhou People’s Hospital, Meizhou City, China

**Keywords:** immune gene, prognosis, Hashimoto’s thyroiditis, papillary thyroid carcinoma, tumor microenvironment

## Abstract

**Background:**

Papillary thyroid carcinoma (PTC) concurrent with Hashimoto’s thyroiditis (HT) was associated with a better clinical prognosis. This study aimed to investigate a potential mRNA gene that affects the development of PTC, which helps PTC concurrent with HT patients have a better prognosis.

**Material/Methods:**

PTC data were obtained from The Cancer Genome Atlas (TCGA) database. And the validation data of tissue specimens were collected from Guangzhou First People’s Hospital. The thyroid tissue sections were hybridized with deleted in malignant brain tumor 1 (DMBT1) probes by situ hybridization. Survival rates were analyzed using Kaplan-Meier curves, and the log-rank test was used to compare group survival rates. Prognosis clinicopathological factors were analyzed by Cox regression. Gene Ontology (GO) and Kyoto Gene and Genomic Encyclopedia (KEGG) pathway enrichment analyses were performed using single-sample gene set enrichment analysis (ssGSEA). Finally, the correlation of deletion in DMBT1 expression with overall immune status, tumor purity, and human leukocyte antigen (HLA) gene expression profile was analyzed.

**Results:**

HT was significantly associated with sex, tumor foci, extrathyroidal extension (ETE), residual tumor, and tumor stage (T stage). Moreover, PTC concurrent with HT had a lower risk of recurrence versus non-HT groups. A total of 136 differentially expressed mRNAs (DEMs) were identified between HT and non-HT groups. Among them, the expression level of DMBT1 in HT groups was statistically higher than that in non-HT groups. A significant association with ETE and recurrence was revealed in the high expression and the low expression of DMBT1. Furthermore, DMBT1 was an independent predictor of survival. The overall immune activity of high expression of DMBT1 was higher than that of the low-expression group.

**Conclusions:**

The PTC patients with HT had better behavior features and prognosis than those with simple PTC. DMBT1 in PTC-HT patients was a potential possible factor that inhibits tumors. High expression of DMBT1 may improve PTC prognosis by immune-related pathways.

## Introduction

Over the past decades, thyroid cancer (TC) has become the most common malignancy of the endocrine organs, with its incidence rising steadily across the world (1). Papillary thyroid cancer (PTC) is the most common pathology type in thyroid cancer, ~90% of thyroid carcinoma (2), and conventional PTC is the main histological variant (3).

Hashimoto’s thyroiditis (HT) is an autoimmune genetic disorder characterized by the destruction of thyroid cells by cell- and antibody-mediated immune responses. In developed countries, HT is the most common cause of hypothyroidism. The estimated incidence of HT is 3.5 per 1000 per year in women and 0.8 per 1000 per year in men.

Dailey et al. (4) first proposed in 1955 that the development of PTC evolved from the development of HT, which many subsequent studies have confirmed. Recent reports indicated that PTC concurrent with HT was associated with better clinical prognosis and less aggressiveness, suggesting that autoimmunity not only was a risk factor for the evolution of TC but also had a protective effect on the further development of the disease (5). Some studies observed that PTC with HT had better clinical behavior features and prognoses. Huang et al. (6) showed that PTC patients with HT had better clinical stage, lower relapse probability, and lower mortality than patients with TC without HT. The average tumor size, distant metastasis, and recurrence probability of the former were significantly lower than those of the latter during the study. The probability of death was 0% during the follow-up of 20 years. Inhwa et al. (7) also suggested that PTC patients with HT had better behavior features and prognoses than did those with single PTC despite frequent multifocality and extrathyroidal extension.

Although the underlying mechanisms for how HT affects PTC is unclear, several hypotheses have been proposed. Among them, inflammation-induced carcinoma has been suggested to be one of the underlying mechanisms (8). In the HT environment, the full activation of inflammatory response, which involves immune cells acting as mediators in chronic inflammation state production, chronic antigenic stimulation may cause neoplastic hyperplasia of the thyroid gland and thus malignant transformation (9, 10). However, this hypothesis is unable to explain why HT has a protective role against PTC progression. In this context, thyroid-specific cytotoxic T cells might play a role in tumor defense-induced autoimmunity (10). Thyroid peroxidase (TPO) and thyroglobulin (Tg) are the main target antigens of cellular immune reactions in HT due to the presence of antigen-presenting cells and thyrocytes, and these immune reactions may lead to target-specific damage of the thyroid gland (10). Because TPO and Tg appear to be target PTC-specific antigens, anti-thyroid antibodies may destroy PTC, in the same way as healthy thyroid gland cells (10, 11). Previous studies have indicated that HT might promote antitumor T cell-mediated immune reactions (12, 13), while other studies have demonstrated that HT was involved in the activation of the apoptotic pathway (14). However, whether or not thyroid-specific cytotoxic T cells recognize TPO and Tg in PTC deserves further investigation.

A genetic predisposition, the protective properties of HT against the progression of PTC has been proposed as another possible mechanism. As an aggressive marker, the BRAF-V600E mutation was less frequently detected in PTC patients concurrent with HT than non-HT (15). The mRNAs have exhibited a great potential in both physiological and pathological processes of PTC (16–18). Thus, dysregulated expression mRNAs may be a promising predictor of poor prognosis in PTC.

Therefore, we hypothesize that PTC occurring with HT is genetically different from simple PTC which means that it may also activate tumor suppressor genes concurrently with HT. The primary aim of the present study was to investigate whether there were differentially expressed mRNAs (DEMs) between PTC concurrent with HT and simple PTC, and the existence of a protective gene; and further explore the potential effect of the immune landscape of mRNAs in PTC.

## Materials and Methods

### Data Collection and Pre-Processing

mRNA expression information and corresponding clinical data of 310 samples, including 42 PTC occurring with HT and 268 simple PTC samples, were obtained from the Cancer Genome Atlas database (TCGA) (16) on October 20, 2019. The validation data of tissue specimens collected from patients undergoing thyroidectomy surgery diagnosed in Guangzhou First People’s Hospital from January 2019 to December 2020, including 26 thyroid carcinoma with HT (HT groups) and 28 cases simple thyroid carcinoma (non-HT groups) tissues, were selected as the research subjects. Moreover, the diagnosis of HT was confirmed through postoperative pathology. The Ethics Committee approved this investigation of Guangzhou First People’s Hospital, and informed consent was obtained from each patient. Also, 54 tissue specimens were used to validate DMBT1 expression between the HT group and the non-HT group. The mRNA that was differentially expressed between PTC-HT and single PTC samples was assessed using the R Studio software program (RStudio version 1.1.463; http://www.r-project.org) and the R package, Limma. |log2FC (fold change) |>2 and P-value <0.05 were considered for subsequent analysis (19). And in the study, *in situ* hybridization was performed to validate the DMBT1 expression obtained from the Guangzhou First People’s Hospital tissue specimens and data, other analysis and results came from the TCGA database.

### *In Situ* Hybridization

The tissue specimens were obtained from Guangzhou First People’s Hospital. According to a previous protocol, the expression level of DMBT1 in tissues was assessed by *in situ* hybridization (ISH). The sequence of the probe were 5′‐TGGAT CCCAA GGACT ACAGA CTACG CTTCA CTGAT‐3′, 5′‐CCACA ATGGC TGGCT CACCC CACCC ACAAC TGTGG CCATA‐3′, 5′‐TACTG GGACA CCAAT GATGC CAATG TGGTT TGCAG‐3′. Briefly, ISH was performed on 5-μm-thick tissue sections from PTC tissues. Then, pepsin, diluted with 3% citric acid, was subsequently used to expose mRNA fragments for 3-30 min. Tissues were then fixed with 4% phosphate-buffered saline for 15 min at room temperature. After pretreatment, tissue sections were hybridized with DMBT probes for 2-4 h. Then, slides were incubated with antibody (#ZN0402; www.bjbalb.com, Beijing, China) at room temperature overnight, and the specific hybridization reaction of the DMBT1-linked probes was observed. The probes were labeled with digoxin on the 3′ and 5′ ends. Finally, the slides were incubated with biotin peroxidase for 30 min at room temperature. The peroxidase reaction was enhanced using 3,3′-diaminobenzidine. To visualize the complete morphology of tissues, the slides were counterstained with hematoxylin and subsequently examined under a bright-field microscope, the Vectra Polaris (Akoya Biosciences, Inc.) (20, 21).

### Analysis of mRNA ISH Slides

The slides expression analysis was diagnosed and recorded by two pathologists of the Department of Pathology, Guangzhou First People’s Hospital, and the expression was scored on 0-10 point scale as follows: 0 points denotes no expression (0), 1-3 points suggests low expression (+), 4-6 points means an intermediate expression (++), and 7-10 points shows high expression (+++).

### Statistical Analysis

Data analysis was performed using SPSS Statistics version 19.0 for Windows (IBM Corp) and the GraphPad Prism software, version 8.1 (www.graphpad.com). The association of HT and each categorical variable was assessed using χ2 tests and Fisher’s exact test when the patient number <5. Multivariate analysis of overall survival (OS) and recurrence-free survival (RFS) was carried out by Cox regression, and hazard ratios (HRs) with 95% CIs were calculated. All P-values were two-sided, and P ≤0.05 was considered significant.

### Survival Analysis

The Kaplan-Meier survival analysis and log-rank test were applied to identify prognostic DEMs. The “survival” package in the R software was used to construct survival curves. And the RFS and OS survival were analyzed by GraphPad Prism software. The survival endpoint was defined as the OS. P <0.05 was considered significant.

### Evaluation of Immune Status

We quantified each PTC sample’s immune cell infiltration level using the immune score evaluated by ESTIMATE (22). Single-sample gene set enrichment analysis (ssGSEA) was used to assess the immune infiltration level of every gene set, for every sample, by calculating separate enrichment scores for each sample and genome. Further, the association between the expression of prognostic DEMs and the immune cells was analyzed.

### The Association Between Immune Genes, Key Immune Checkpoints, and the Expression Level of Prognostic DEMs Was Analyzed

The Mann-Whitney U test was used to compare the immune score differences of the expression level of prognostic DEMs (high and low).

### Functional Enrichment, KEGG, and GO Analyses

Gene Ontology (GO) functional annotation and Kyoto Encyclopedia of Genes and Genomes (KEGG) pathway enrichment were performed in R using the “clusterProfiler” package, and p. adjust (FDR) < 0.05 was considered statistically significant (23).

## Results

### Clinicopathologic Features of Patients With PTC Coincident With HT and Simple PTC

**Figure 1:**
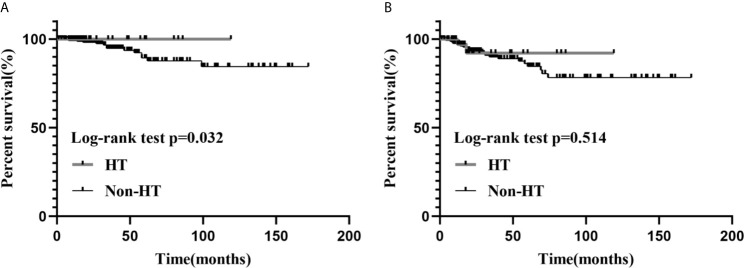
The Kaplan–Meier survival analysis of RFS **(A)** and OS **(B)**, between the HT and the non-HT groups.

Median follow-up for all patients was 25 months (IQR, 14-53 months). Median follow-up for patients in the HT group was 17.5 months (IQR, 12.8-40.5 months), during which time there were two cases of recurrence and no case mortalities. Median follow-up was 25.5 months (IQR, 14-54.8 months) in the non-HT group, during which time there were 25 cases of recurrence and 14 cases of mortality. Our univariate analyses demonstrated a significant association between HT and patient’s sex (P<0.001), tumor foci (P=0.007), ETE (gross) (P=0.001), residual tumor (P=0.045), and T stage (P=0.006) (**Table 1**). Furthermore, Kaplan–Meier curves showed that the HT group had a low risk of RFS versus the non-HT group (P=0.032) (**Figure 1**).

**Table 1:** Relationship between HT and clinicopathological parameters.

Patients’ parameters	Total (PTC)	Non-HT(268)	HT(42)		Odds ratio (95%CI)	*P* value
**Age**						
<55	223	190		33	1	0.303
≥55	87	78		9	0.664(0.304-1.453)	
**Sex**						
Women	208	198		10	1	<0.001*
Men	102	70		32	1.131(0.529-2.420)	
**Ethnicity category**						
White	221	192		29	1	0.297
Asian	29	26		3	0.151(0.077-0.295)	
Black	18	18		0	1.094(1.049-1.140)	
**Tumor foci**						
Unifocality	185	167		18	1	0.007*
Multifocality	123	100		23	2.453(1.265-4.758)	
**Histology**						
CPTC	235	204		31	1	0.071
FVPTC	53	42		11	1.724(0.803-3.699)	
TCPTC	22	22		0	1.108(1.061-1.156)	
**ETE (gross)**						
No	203	166		37	1	0.001*
Yes	102	98		4	0.183(0.063-0.529)	
**Residual tumor**						
No	246	210		36	1	0.045*
Yes	37	36		1	0.162(0.022-1.219)	
**Recurrence**						
No	283	243		40	1	0.554
Yes	27	25		2	0.486(0.111-2.132)	
**Mortality**						
No	296	254		42	1	1.000
Yes	14	14		0	0.827(0.181-3.769)	
**T stage**						
1	78	63		15	1	0.006*
2	112	91		21	0.969(0.464-2.024)	
3	102	96		6	0.262(0.097-0.713)	
4	18	18		0	1.286(1.144-1.444)	
**N stage**						
0	127	103		24	1	0.101
1	152	134		18	0.576(0.297-1.119)	
**M stage**						
M0	186	155		31	1	1.000
M1	13	11		2	0.909(0.192-4.305)	
**AJCC stage**						
I	247	214		33	1	0.126
II	48	41		7	1.107(0.459-2.673)	
III	11	11		0	1.051(1.021-1.083)	
IV	4	2		2	1.179(0.250-5.558)	

HT, Hashimoto’s thyroiditis; PTC, papillary thyroid cancer; CPTC, conventional papillary thyroid cancer; FVPTC, follicular variant papillary thyroid cancer; TCPTC, tall cell variant papillary thyroid cancer; ETE, extrathyroidal extension; T, tumor size; N, lymph node; M, metastasis; AJCC, 8th edition American Joint Committee on Cancer staging. *P < 0.05; CI, confidence intervals.

### DEMs in PTCs Occurrence With HT Group *Versus* Non-HT Groups

**Figure 2:**
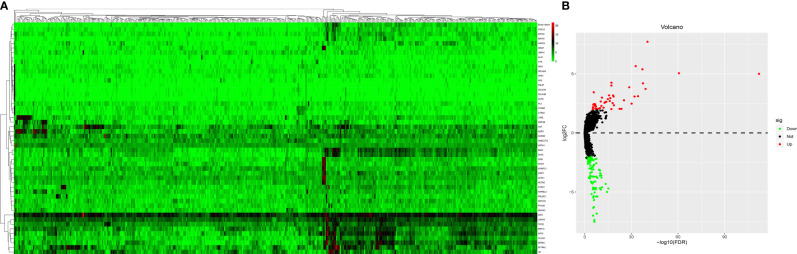
Heatmap analysis **(A)** and a volcano plot **(B)** of differentially expressed mRNAs. The gene expression pattern depends on the differentially expressed mRNAs. Supervised clustering of papillary thyroid carcinomas (PTCs) did exhibit a significant clustering effect between the PTC-HT group and the non-HT group. Each cell in the matrix represents the expression level of a gene feature in an individual pattern. Red or green color reflects a relative upregulation or downregulation, respectively, as indicated in the scale bar.

The mRNA expression profiles between HT and non-HT groups from the TCGA database were downloaded and analyzed. |logFC(fold change)| >2 and a P-value <0.05 were considered as the cut-off threshold to screen out DEMs. A total of 39 upregulated and 97 downregulated DEMs were identified between the HT and non-HT groups. The volcano plot and heatmap plot of the related DEMs in PTCs are presented in **Figure 2**. The top 10 upregulated and downregulated DEMs are listed in **Table 2**.

**Table 2:** The top 10 upregulated and downregulated differentially expressed mRNAs.

Gene symbol	LogFC	FDR	Change
ONECUT2	2.846598178	4.00E-37	Up
AKR1C2	3.003495786	3.94E-33	Up
PTH2R	3.009139202	2.19E-31	Up
SFTPA2	4.127557267	3.07E-23	Up
VGF	3.699246144	1.10E-19	Up
VIL1	2.868695432	2.25E-18	Up
HMP19	2.425403134	1.64E-16	Up
NTRK1	3.413005	7.53E-16	Up
HP	3.035935495	5.81E-13	Up
SAA1	2.786086348	4.95E-12	Up
MATN1	-5.005607624	3.08E-09	Down
ACTA1	-4.564569023	4.18E-08	Down
GDF6	-4.817671822	8.32E-08	Down
MYBPC1	-4.171266076	1.68E-07	Down
CKM	-5.067448813	3.43E-07	Down
NKAIN1	-4.1177435	9.82E-07	Down
LHX2	-4.803160418	1.85E-06	Down
ACTN2	-3.692894621	2.17E-06	Down
KIRREL3	-3.522301643	3.24E-06	Down
MYH7	-5.033045481	3.25E-06	Down

FC, fold change; FDR, false discovery rate.

### Survival Analysis

**Figure 3:**
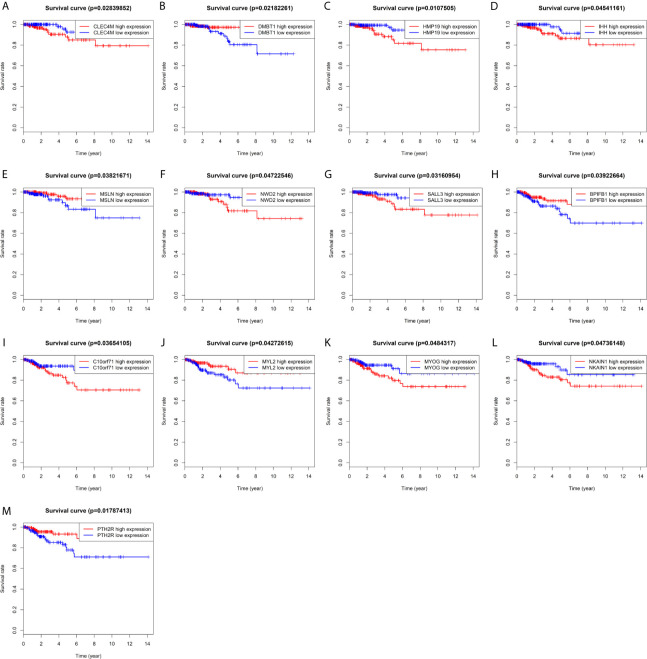
Prognostic significance of differentially expressed mRNAs. **(A)** CLEC4M; **(B)** DMBT1; **(C)** HMP19; **(D)** IHH; **(E)** MSLN; **(F)** NWD2; **(G)** SALL3; **(H)** BPIFB1; **(I)** C10or71; **(J)** MYL2; **(K)** MYOG; **(L)** NKAIN1; **(M)** PTH2R.

Kaplan-Meier univariate survival analyses were performed in the present study to investigate the impact of clinical information and gene expression profiles on RFS and OS. Among all the 126 DEMs examined in this study, six mRNAs, including BPIFB1, C10orf71, MYL2, MYOG, NKAIN1, and PTH2R, were closely associated with RFS in patients with PTC (**Figures 3H–M**), and seven mRNAs including CLEC4M, DMBT1, HMP19, IHH, MSLN, NWD2, and SALL3 were closely related to OS in patients with PTC (P<0.05; **Figures 3A–G**). Among them, high expression of five mRNA (DMBT1, MSLN, BPIFB1, MYL2, PTH2R) had a lower risk of survival and recurrence (**Figures 3B, E, H, J, M**).

### Expression Levels of DMBT1, MSLN, BPIFB1, MYL2, and PTH2R in HT and Non-HT Groups

**Figure 4:**
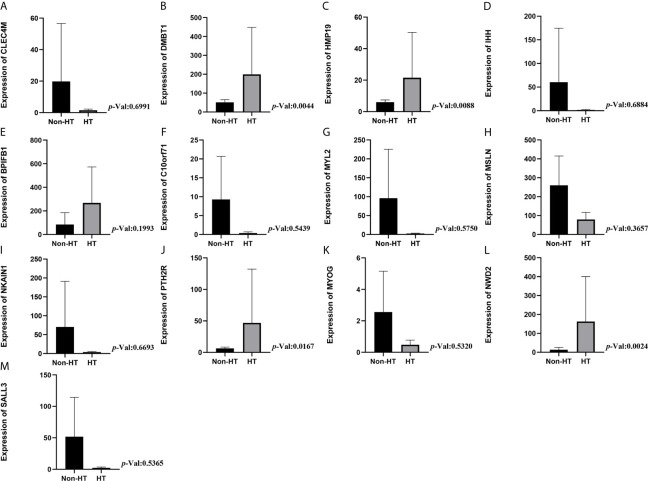
Expression levels of mRNA in HT and non-HT groups. **(A)** CLEC4M; **(B)** DMBT1; **(C)** HMP19; **(D)** IHH; **(E)** MSLN; **(F)** NWD2; **(G)** SALL3; **(H)** BPIFB1; **(I)** C10or71; **(J)** MYL2; **(K)** MYOG; **(L)** NKAIN1; **(M)** PTH2R.

The expression level of DMBT1 and PTH2R in the HT group was statistically higher than that in the non-HT group (P<0.004 and P=0.017, respectively, **Figures 4B, J**). There was no statistically significant difference in the expression level of BPIFB1, MSLN, and MYL2 in the HT group (P=0.199, P=0.366, and P=0.575, respectively, **Figures 4E, H, G**). Additionally, there was no statistically significant difference in the expression level of the other 8 prognostic genes in the HT group (**Figures 4A, C, D, F, I, K–M**).

### The Validation of DMBT1 by the Data of *In Situ* Hybridization

**Figure 5:**
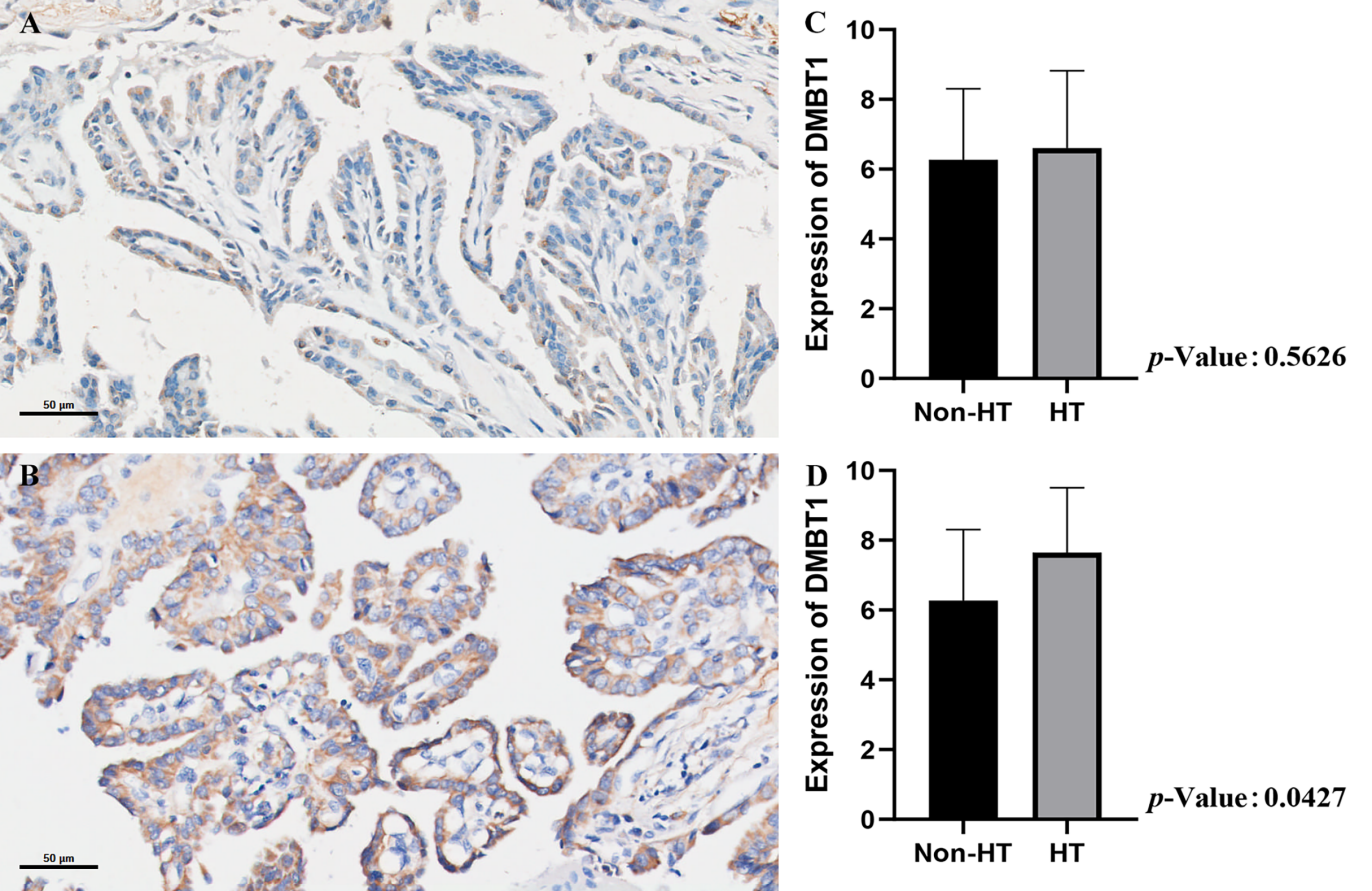
The validation of DMBT1 by the data of *in situ* hybridization. **(A)** PTC concurrent with non-HT. **(B)** PTC concurrent with HT. **(C)** The expression level of the non-HT group *vs.* HT group. **(D)** The expression level of the non-HT group *vs.* HT group (TPOAb>100 IU/ml).

The results of the HT group revealed that the intensity and extent of hybridization signals of DMBT1 in TC tissues were significantly higher than those in non-HT group tissues, particularly in TPOAb >100 (HT group) (**Figures 5A–D** and **Table 3**).

**Table 3:** The validation of DMBT1 by the data of *in situ* hybridization.

Expression of DMBT1	Non-HT	HT		*P* value	HT (TPOAb>100 IU/ml)	*P* value
**+++(7-10)**	14		17	0.5626	10	0.0427
**++(4-6)**	10		7		5	
**+(1-3)**	2		1		1	
**0**	2		1		0	

HT, Hashimoto’s thyroiditis. The P value was the result of numerical variables by T test.

### Relationship Between the Expression Levels of DMBT1 and the Clinicopathological Features of Patients With PTC

Between the high and low expression of DMBT1, our univariate analyses demonstrated a significant association between patient’s ETE (gross) (P=0.045), recurrence (P=0.023), N stage (P=0.008), and AJCC stage (P<0.001). The results are detailed in **Table 4**.

**Table 4:** Relationship between the expression levels of DMBT1 and clinicopathological parameters.

Patients’ parameters	Total	Expression of DMBT1	P value
**Age**			
<55	223	72.82	0.887
≥55	87	67.17	
**Sex**			
Women	230	76.58	0.613
Men	80	55.86	
**Ethnicity category**			
White	221	76.47	0.121
Asian	29	55	
Black	18	80.83	
**Tumor foci**			
Unifocality	185	52.3	0.198
Multifocality	123	99.25	
**Histology**			
CPTC	235	86.02	0.143
FVPTC+TCPTC	75	24.88	
**Lymph nodes positivity (>5)**			
No	116	43.87	0.179
Yes	125	103.6	
**ETE (gross)**			
No	203	79.11	0.045*
Yes	102	58.56	
**Residual tumor**			
No	246	73.95	0.786
Yes	37	58.3	
**Recurrence**			
No	283	74.28	0.023*
Yes	27	39.26	
**Mortality**			
No	296	72.04	0.836
Yes	14	54.14	
**T stage**			
I-II	190	86.25	0.291
III-IV	120	47.46	
**N stage**			
0	127	91.63	0.008*
1	152	52.01	
**M stage**			
M0	186	77.62	0.773
M1	13	109.2	
**AJCC stage**			
I-II	295	145.12	<0.001*
III-IV	15	74.13	

CPTC, conventional papillary thyroid cancer; FVPTC, follicular variant papillary thyroid cancer; TCPTC, tall cell variant papillary thyroid cancer; ETE, extrathyroidal extension; T, tumor size; N, lymph node; M, metastasis; AJCC, 8th edition American Joint Committee on Cancer staging. *P < 0.05.

### Recurrence and Survival Risk Factors

Multiple regression analyses were conducted, controlling for HT, age (55 years), DMBT1, sex, ethnicity category, tumor foci, ETE (gross), histology, residual tumor, T stage, N stage, M stage, and AJCC stage. Our analyses found that only DMBT1 (P=0.043) and N stage (P=0.046) were independent predictors of recurrence. Moreover, age (55 years) (P=0.049), DMBT1 (P=0.032), residual tumor (P=0.046), and T stage (P=0.003) were independent predictors of survival. The results are detailed in **Table 5**.

**Table 5:** Cox multivariate regression analyses of factors associated with recurrence (and survival).

Clinicopathologic features	RFS		OS	
	HR (95% CI)	P	HR (95% CI)	P
HT	0.563 (0.058-5.490)	0.621	0.042 (0.000-77.786)	0.408
Age (≥55)	0.669 (0.139-3.230)	0.617	312.767 (1.034-94582.626)	0.049*
DMBT1 (high)	0.947 (0.327-2.741)	0.043*	0.903 (0.202-4.040)	0.032*
Male sex	0.402 (0.065-2.479)	0.326	1.899 (0.635-5.682)	0.251
Ethnicity category (non-White)	1.492 (0.489-4.552)	0.482	0.119 (0.002-8.878)	0.333
Multifocality	1.837 (0.408-8.264)	0.428	0.169 (0.022-1.301)	0.088
ETE	0.437 (0.043-4.408)	0.482	1.803 (0.621-5.230)	0.278
Histology (non-CPTC)	1.416 (0.479-4.191)	0.529	0.392 (0.060-2.575)	0.329
Residual tumor	2.158 (0.338-13.787)	0.416	3.516 (1.022-12.100)	0.046*
T stage	3.262 (0.625-17.032)	0.161	2.751 (1.400-5.406)	0.003*
N stage	2.559 (1.015-6.450)	0.046*	2.269 (0.601-8.559)	0.227
M stage	1.500 (0.196-11.478)	0.696	–	0.735
AJCC stage	2.123 (0.525-8.581)	0.291	0.871 (0.305-2.485)	0.797

HR, hazard ratios; CI, confidence intervals; RFS, recurrence-free survival; OS, overall survival; ETE, extrathyroidal extension; CPTC, conventional papillary thyroid cancer; T, tumor size; N, lymph node; M, metastasis; AJCC, 8th edition American Joint Committee on Cancer staging. *P < 0.05.

### Evaluation of Immune Status Between the High Expression and Low Expression of DMBT1

**Figure 6:**
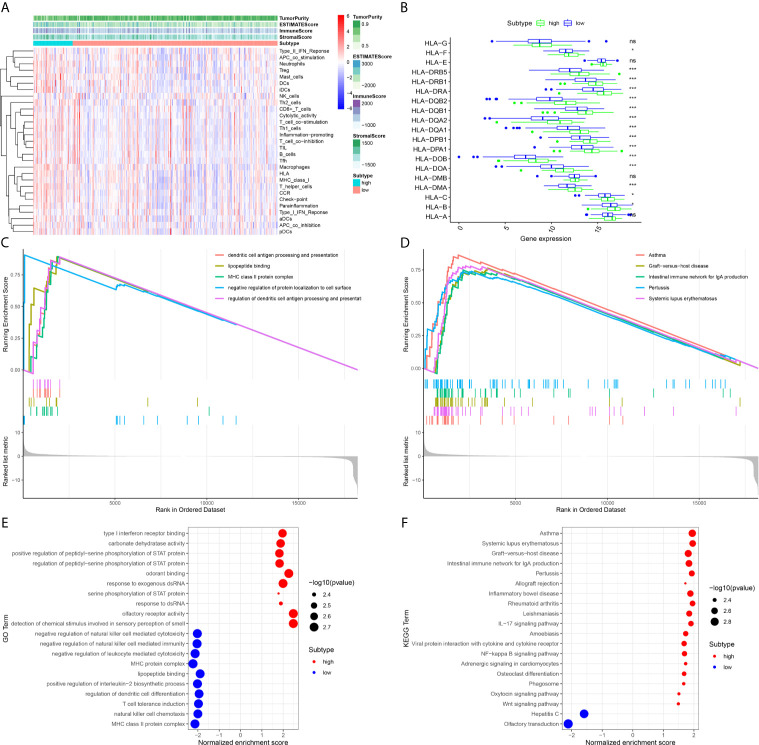
The immune-related analysis of the high expression and low expression of DMBT1 was analyzed by ssGSEA and ESTIMATE methods. **(A)**. The heatmap of overall immune status and tumor purity in high-expression versus low-expression DMBT1 groups. **(B)**. HLA gene expression profiles in high-expression versus low-expression DMBT1 groups. **(C)**. Gene Ontology (GO)-enriched pathways of patients with high TIM risk scores. **(D)**. The Kyoto Encyclopedia of Genes and Genomes (KEGG)-enriched pathways of patients with high TIM risk scores. **(E)**. GO functional annotation pathway enrichments in high-expression versus low-expression DMBT1 groups. **(F)**. KEGG pathway enrichments in high-expression versus low-expression DMBT1 groups. (*P < 0.05, ***P < 0.001, ns P > 0.05).

To further explore the association between the expression level of DMBT1 and the immune system, the ssGSEA method was used to estimate the overall immune status of the high expression and low expression of DMBT1 by analyzing the expression profiles of the 29 immune signature gene sets (**Figure 6A**). The ssGSEA results demonstrated that tumor purity of the high expression of DMBT1 in TCGA was significantly higher than that of the low expression of DMBT1, which suggested that infiltrating stromal/immune cells existed in the TME of high-expression DMBT1 samples (**Figure 6A**). The human leukocyte antigen (HLA) plays a key role in immune regulation and autoimmune disease etiology. The analysis showed that the expression of key HLA genes except HLA-G, HLA-E, HLA-DMA, and HLA-A in the high expression of DMBT1 was significantly higher than those in the low expression of DMBT1 (**Figure 6B**).

### Functional Enrichment, KEGG, and GO Analyses

To investigate the underlying mechanisms of the prognostic effects of DMBT1, ssGSEA tests were performed. Our results demonstrated that the high expression of DMBT1 was linked with the activation of pathways regulating immune process and tumor progression in GO analysis (**Figure 6E**). Moreover, the results of KEGG analysis demonstrated that the DMBT1 gene was correlated with immune-related diseases, such as asthma, graft versus host disease, the intestinal immune network for IgA production, pertussis, and systemic lupus erythematosus (**Figure 6F**), and the total ssGSEA enrichment score (**Figures 6C, D**).

### The Analysis of the Association Between Key Genes and the Expression Level of DMBT1

**Figure 7:**
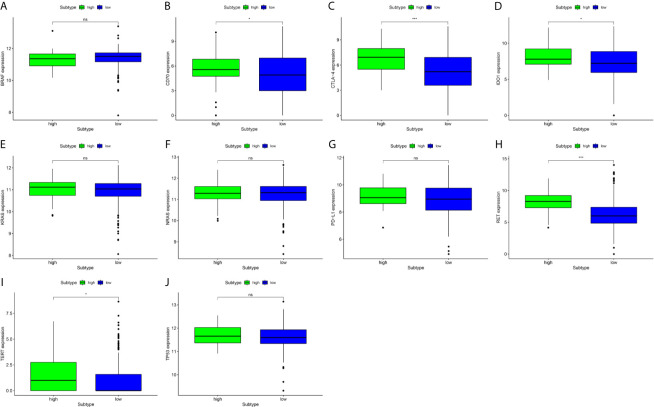
Gene expression analysis of key prognosis gene checkpoint in high-expression versus low-expression DMBT1 groups. **(A)** BRAF; **(B)** CD70; **(C)** CTLA-4; **(D)** IDO1; **(E)** KRAS; **(F)** NRAS; **(G)** PDL-1; **(H)** RET; **(I)** TERT; **(J)** TP53. (*P < 0.05, ***P < 0.001, ns P > 0.05).

We found that there was significant heterogeneity among the immune-infiltrating patterns of PTC patients. BRAF, CD-70, CTLA-4, IDO1, KRAS, NRAS, PD-L1, RET, TERT, and TP53 mRNA expression levels were compared in high-expression and low-expression DMBT1 (**Figures 7A–J**). We found that the mRNA expression levels of CD-70, CTLA-4, IDO1, RET, and TERT in the high-expression DMBT1 groups were higher than those in the low-expression DMBT1 groups (**Figures 7B–D, H, I**), of which CTLA-4 and IDO1 were the key immune checkpoint genes (**Figures 7C, D**) (P < 0.05).

## Discussion

The association of PTC with HT has remained an active focus of research and controversy since it was first described in the 1950s. In the first step, our study was to verify the association between PTC and HT by evaluating two groups of PTC patients (HT and non-HT groups). Our studies demonstrated that HT was significantly associated with sex, tumor foci, ETE (gross), residual tumor, and T stage, which were the major factors influencing the better prognosis of PTC by the OR value. Furthermore, the RFS and OS curves demonstrated that the HT group exhibited a relative higher survival rate versus the non-HT group, and the former group had a better prognosis. These data are similar to the results reported previously (5, 24). It was found that the coexistence of PTC and HT was negatively associated with the presence of ETE, lymph node metastasis, distant metastasis, and recurrence. Therefore, we believe that PTC concurrent with HT presents more favorable clinicopathologic characteristics and a better prognosis than simple PTC patients.

Although the underlying mechanism of how HT affects PTC remains unclear, some hypotheses have been proposed. Among them, inflammation-induced carcinoma has been regarded as one of the possible mechanisms (9). A genetic predisposition, the protective properties of HT against the progression of PTC has been proposed as another possible mechanism. As an aggressive marker, the BRAF-V600E mutation was less frequently detected in PTC patients concurrent with HT than non-HT (15). In the context of autoimmune thyreopathy, the thyroid has high concentrations of TG, TTF-1, HBM1, galectine3, and CK-19 (25). And Unger et al. (26) revealed that PTC patients concurrent with HT had a high expression of protein p63 versus normal thyroid glands or concurrent with nodular goiter or follicular adenomas.

In the second step, we further explored whether some genes promote a better prognosis in PTC patients concurrent with HT than without HT. The present study further demonstrated that 39 upregulated and 97 downregulated DEMs were identified between the HT and non-HT groups. Among these genes, we found a significantly high expression of DMBT1 in the HT group compared with the non-HT group. Several previous studies elucidated that DMBT1 was obviously expressed in normal tissues compared with cancer or disease tissues (27–37).

DMBT1 is located on the long arm of chromosome 10 (38), and its sequence mainly consists of repeated high homologous exons and introns (39). Some research has reported that tumors like esophageal carcinoma (40), colon carcinoma (41), bladder carcinoma (42), breast cancer (31), prostate carcinoma (31), and non-small cell carcinoma (27) presented a lower expression of DMBT1 compared with normal tissues. Therefore, the DMBT1 gene may be able to inhibit the progression of the tumor. According to Mollenhauer and his team (30), DMBT1 might play an important role in the potential function of human health. In an underlying mechanism, the high expression of the DMBT1 gene may inhibit the occurrence of tumors by promoting cell differentiation because the formation of tumors can lead to cell disorders. Moreover, some studies demonstrated that DMBT1 was associated with immune defense, cell polarization, differentiation and regeneration, autoimmune disease, and Crohn’s disease (33, 35, 36).

Further, the relationship between the expression levels of DMBT1 and the clinicopathological features of PTCs was analyzed. The results showed that the expression of DMBT1 in PTC was significantly correlated with a lower risk of ETE (gross) and recurrence, lymph nodes metastases, and AJCC stage. According to the TNM staging system recommended by the 8th edition of AJCC, ETE, incomplete tumor resection, tumor size, lymph node metastases, and distant metastases are the risk factors of recurrence and mortality (43). This finding was similar to previous studies (32) where the DMBT1 gene was associated with a lower risk of clinical staging, lymph node metastasis, and pathological type and size of tumor, which have suggested that highly expressed DMBT1 possibly inhibits PTC.

These results may further support the idea that DMBT1 acts as a tumor suppressor gene in the process of PTC. Moreover, the studies demonstrated that the high-expression groups of DMBT1 might decrease the risk of tumor recurrence in PTC patients. Also, the multivariate regression analysis of the data revealed that DMBT1 was an independent prognostic factor for predicting recurrent disease and mortality. These results are further supported by our research on the role of the tumor suppressor gene in the high-expression groups of DMBT1 in suppressing cancer.

DMBT1 is a protective gene, which has been confirmed by many studies. DMBT1 is associated with innate immunity and autoinflammatory enteritis (Crohn’s disease). Moreover, prior studies in mice have reported that DMBT1-specific immune responses lead to interstitial lung disease (autoimmune syndromes), providing substantial evidence that the autoreactivity of targeting DMBT1 was pathogenic in a subset of human interstitial lung disease patients (44). To further study the potential molecular mechanism of DMBT1, GSEA was performed. The results show that the expression changes of DMBT1 were related to immune regulation, which provides clues for further research. To explore the influence of DMBT1 expression on the PTC tumor microenvironment (TME), ssGSEA and the ESTIMATE method were used to assess the overall immune status and tumor purity in the PTC TME.

Interestingly, the overall immune activity of the high expression DMBT1 was higher than that of the low-expression group. Correspondingly, the tumor purity of the high expression of DMBT1 was lower than that of the low expression of DMBT1, suggesting that more stromal cells and immune cells were infiltrated in the TME. The expression of HLA analysis also demonstrated that vital HLA genes in the high expression of DMBT1 were highly expressed, suggesting that local immune regulation and response were more active, which partly supported the results that PTC patients with HT had better behavior features and prognosis than those with simple PTC. Moreover, the key immune checkpoint genes, including many established or potential immunotherapeutic targets such as CTLA-4 and IDO1, were significantly different between the high and low expression levels of DMBT1. The above results demonstrated that the high expression of DMBT1 might improve PTC patients’ prognosis by immune-related pathways.

The present study included some limitations. Firstly, the mRNA expression data used were downloaded from a single database (the TCGA database) instead of multiple databases. The ISH method has significant limitations, in which the DMBT1 gene was significantly degraded in paraffin-embedded tissue specimens. Secondly, several data were omitted from various clinicopathological characteristics, and only 42 PTC concurrent with HT patients took part. Thirdly, this study is a retrospective analysis using TCGA data, prospective clinical trials are required to provide more reliable results. Finally, the diagnostic criteria for HT are slightly different since the TCGA data come from multiple centers.

## Conclusions

The HT patients with PTC had better behavior features and prognosis than those with simple PTC. DMBT1 in PTC-HT patients was a potential factor that inhibits tumors. High expression of DMBT1 may improve PTC prognosis by immune-related pathways.

## Data Availability Statement

The datasets presented in this study can be found in online repositories. The names of the repository/repositories and accession number(s) can be found in the article/supplementary material.

## Ethics Statement

The studies involving human participants were reviewed and approved by Ethics Committee of Guangzhou First People’s Hospital. The patients/participants provided their written informed consent to participate in this study. Written informed consent was obtained from the individual(s) for the publication of any potentially identifiable images or data included in this article.

## Author Contributions

Conception: X-xG and BX. Design and revision of the manuscript: X-xG, J-hF, and FS. Analysis and interpretation of data: X-xG, FS, J-hF, W-sC, Y-yL, S-jL, Y-qL, and S-sM. All authors contributed to the article and approved the submitted version.

## Funding

This research was supported by Guangzhou Medicine and Health Care Technology Projects (20211A011010).

## Conflict of Interest

The authors declare that the research was conducted in the absence of any commercial or financial relationships that could be construed as a potential conflict of interest.

## Publisher’s Note

All claims expressed in this article are solely those of the authors and do not necessarily represent those of their affiliated organizations, or those of the publisher, the editors and the reviewers. Any product that may be evaluated in this article, or claim that may be made by its manufacturer, is not guaranteed or endorsed by the publisher.
